# Simvastatin combined with antiviral therapy for hepatitis B cirrhosis with portal hypertension: A case report

**DOI:** 10.1097/MD.0000000000046933

**Published:** 2026-01-02

**Authors:** Haodong Zhang, Jinjin Li, Yong Zhou, Xuefang Li, Hui Wang, Wei Gou, Zhensheng Liu, Xiaolong Qi

**Affiliations:** a Department of Metabolic Hepatology, Qingdao Public Health Clinical Center, Qingdao City, China; b Southeast University Affiliated Zhongda Hospital, Nanjing City, China.

**Keywords:** hepatic venous pressure gradient, hepatitis B cirrhosis, portal hypertension, simvastatin

## Abstract

**Rationale:**

Clinical management of hepatitis B cirrhosis complicated with portal hypertension remains challenging. nonselective beta-blockers like carvedilol, first-line agents, have notable limitations. While simvastatin may improve liver fibrosis and reduce portal hypertension, clinical evidence – especially regarding hepatic venous pressure gradient (HVPG) – is scarce. This study investigates the efficacy and safety of simvastatin combined with antiviral therapy in patients intolerant to carvedilol.

**Patient concerns:**

Case 1: A 54-year-old male was admitted due to abnormal liver function for 2 weeks and fatigue with abdominal distension for 2 days. Vital signs were stable, with chronic liver disease facies, soft abdomen, positive hepatic percussion tenderness, no hepatosplenomegaly, and no ascites. Case 2: A 62-year-old male presented with intermittent fatigue for 3 months (aggravated for 1 week). His pulse rate was 49 beats per minute with mild dizziness (no chest tightness/pain), accompanied by chronic liver disease facies, liver palms, soft abdomen, positive hepatic percussion tenderness, and no ascites or bleeding.

**Diagnoses:**

Case 1: Compensated hepatitis B cirrhosis; Portal hypertension; Carvedilol-induced bradycardia. Case 2: Compensated hepatitis B cirrhosis; Portal hypertension; Sinus bradycardia.

**Interventions:**

Basic treatment focused on HBV suppression, with portal hypertension management adjusted per contraindications and responses. Case 1 initially received carvedilol but switched to long-term simvastatin due to bradycardia. Case 2 was prescribed simvastatin upfront (given preexisting bradycardia); after self-discontinuing (HVPG increased), simvastatin was restarted for long-term maintenance.

**Outcomes:**

Nucleoside analogues (antiviral) plus simvastatin (reducing intrahepatic vascular resistance) effectively lowered portal venous pressure without drug-related adverse reactions.

**Lessons:**

For hepatitis B cirrhosis patients with portal hypertension who are intolerant to or have contraindications for carvedilol, simvastatin combined with antiviral therapy is a safe, effective alternative. It significantly reduces HVPG, improves liver stiffness, and causes no obvious adverse events.

## 1. Introduction

The management of portal hypertension in hepatitis B cirrhosis remains a clinical challenge. Although the Baveno VII consensus recommends nonselective beta-blockers (NSBBs) such as carvedilol as first-line options,^[1]^ safety concerns with NSBBs persist in patients with refractory ascites or spontaneous bacterial peritonitis.^[2]^ Additionally, patients with second- or third-degree atrioventricular block (without a pacemaker), asthma, or sinus bradycardia represent absolute contraindications for NSBB use,^[3]^ posing significant clinical limitations. While emerging studies suggest simvastatin may have beneficial effects on liver fibrosis and portal hypertension,^[4–6]^ limited clinical data are currently available. Here, we report two cases of hepatitis B-related cirrhotic portal hypertension effectively treated with the addition of simvastatin to antiviral therapy, in hopes of contributing to the research on pharmacological treatments for cirrhotic portal hypertension.

## 2. Case presentation

### 2.1. Case 1

A 54-year-old male was admitted due to “abnormal liver function for 2 weeks and fatigue with abdominal distension for 2 days.” Two weeks prior, routine physical examination revealed abnormal liver function, and 2 days before admission, he developed fatigue and abdominal distension without obvious precipitating factors. On admission, vital signs were: temperature 36.3°C, pulse 70 beats per minute, blood pressure 120/78 mm Hg. He was alert, in fair general condition, with a chronic liver disease facies, no jaundice in the skin or sclera, soft abdomen without tenderness or rebound pain, positive hepatic percussion tenderness, no hepatosplenomegaly on palpation, and negative shifting dullness.

#### 2.1.1. Laboratory and imaging findings

HBV DNA: 2.63 × 10⁷ IU/mL; HBsAg: 3291.45 IU/mL; anti-HBs: 0 mIU/mL; HBeAg: 401.88 s/co; anti-HBe: 23.79 s/co; anti-HBc: 6.60 s/co.

Liver function: total bilirubin 23.5 μmol/L, direct bilirubin 6.9 μmol/L, indirect bilirubin 16.6 μmol/L, ALT 96 U/L, AST 47 U/L, GGT 58 U/L.

Renal function: UREA 4.6 mmol/L, CREA 65 µmol/L.

Fasting blood glucose: 4.9 mmol/L.

Myocardial enzyme profile: LDH 122 U/L, CK 53 U/L, CKMB 6 U/L, HBDB 108 U/L.

Abdominal ultrasound: consistent with cirrhosis; transient elastography: CAP 214 dB/m, liver stiffness measurement (LSM) 21.9 kPa.

#### 2.1.2. Treatment course

Upon admission, antiviral therapy with tenofovir alafenamide and liver-protective and enzyme-lowering therapy with glycyrrhizinic acid monoammonium cysteine sodium chloride injection were initiated. On day 8 of admission, hepatic venous pressure gradient (HVPG) was measured via antecubital vein catheterization, showing HVPG = 12.9 mm Hg. Carvedilol (6.25 mg orally once daily) was prescribed to reduce portal pressure. After 7 days, the patient’s heart rate dropped to 48 to 55 beats per minute (a significant decrease from baseline), prompting discontinuation of carvedilol. Simvastatin (10 mg orally once daily) was substituted for continued treatment.

#### 2.1.3. Seven-month follow-up

Virological and serological parameters: HBV DNA 5.67 × 10^2^ IU/mL; HBsAg 4336.06 IU/mL; anti-HBs 0.11 mIU/mL; HBeAg 21.58 s/co; anti-HBe 2.28 s/co; anti-HBc 7.93 s/co.

Liver function: total bilirubin 20.6 μmol/L, direct bilirubin 8.9 μmol/L, indirect bilirubin 11.7 μmol/L, ALT 36 U/L, AST 25 U/L, GGT 42 U/L, albumin 40.8 g/L.

Renal function: UREA 5.7 mmol/L, CREA 66.4 µmol/L.

Fasting blood glucose: 5.43 mmol/L.

Myocardial enzyme profile: LDH 159 U/L, CK 90 U/L, CKMB 10.5 U/L, HBDB 118 U/L.

**Figure 1.**
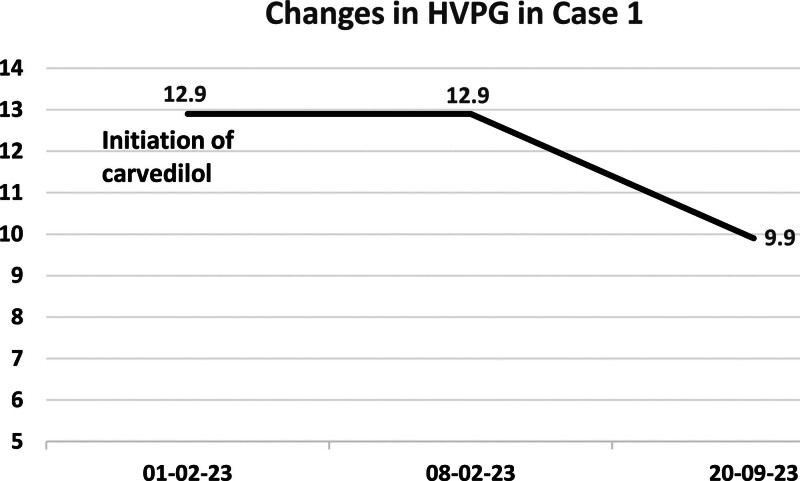
Changes in HVPG in case 1. HVPG = hepatic venous pressure gradient.

Imaging: abdominal ultrasound unchanged (cirrhosis); transient elastography: CAP 211 dB/m, LSM 14.2 kPa; repeat HVPG measurement: 9.9 mm Hg, representing a 23.2% reduction from baseline (dynamic changes in HVPG shown in Fig. 1).

We continued to follow up with the patients after the second HVPG measurement, and the patients underwent reexamination of relevant indicators 4 months later.

Virological and serological parameters: HBV DNA <10^2^ IU/mL; HBsAg 3109.33 IU/mL; anti-HBs 0.31 mIU/mL; HBeAg 3.2 s/co; anti-HBe 1.13 s/co; anti-HBc 6.81 s/co.

Liver function: total bilirubin 20.8 μmol/L, direct bilirubin 7.9 μmol/L, indirect bilirubin 12.9 μmol/L, ALT 29 U/L, AST 26 U/L, GGT 32 U/L, albumin 45.1 g/L.

Renal function: UREA 4.65 mmol/L, CREA 71.2 µmol/L.

Fasting blood glucose: 5.81 mmol/L.

Myocardial enzyme profile: LDH 155 U/L, CK 96 U/L, CKMB 9.4 U/L, HBDB 117 U/L.

Imaging: abdominal ultrasound unchanged (cirrhosis); transient elastography: CAP 176 dB/m, LSM 8.4 kPa (laboratory changes listed in Table 1).

**Table 1:** Changes in laboratory findings of case 1.

	Laboratory tests before the first HVPG measurement	Laboratory tests before the second HVPG measurement	4 mo after the end of follow-up
HBsAg (IU/mL)	3291.45	4336.06	3109.33
HBsAb (IU/mL)	0	0.11	0.31
HBcAb (s/co)	6.6	7.93	6.81
HBeAg (s/co)	401.88	21.58	3.20
HBeAb (s/co)	23.79	2.28	1.13
HBV-DNA (IU/mL)	2.63 × 107	5.67 × 102	<102
TBIL (µmol/L)	23.5	20.6	20.8
DBIL (µmol/L)	6.9	8.9	7.9
ALT (U/L)	96	36	29
AST (U/L)	47	25	26
ALP (U/L)	64	83	108
GGT (U/L)	58	42	32
ALB (g/L)	37	40.8	45.1
UREA (mmol/L)	4.6	5.7	4.65
CREA (µmol/L)	65	66.4	71.2
GLU (mmol/L)	4.9	5.43	5.81
LDH (U/L)	122	159	155
CK (U/L)	53	90	96
CKMB (U/L)	6	10.5	9.4
HBDB (U/L)	108	118	117
CHOL (mmol/L)	5.3	4.07	4.18
HDL (mmol/L)	1.57	1.68	1.51
LDL (mmol/L)	2.91	2.1	2.35
TG (mmol/L)	0.83	0.62	0.85
WBC (109/L)	3.66	3.74	4.22
N (109/L)	1.99	1.76	2.58
RBC (1012/L)	4.53	4.60	4.88
PLT (109/L)	137	165	187
CAP	214	211	176
E	21.9	14.2	8.4

ALB = albumin, ALP = alkaline phosphatase, ALT = alanine aminotransferase, AST = aspartate aminotransferase, CAP = controlled attenuation parameter, CHOL = total cholesterol, DBIL = direct bilirubin, E = liver stiffness measurement, GGT = gamma-glutamyl transferase, HBcAb = hepatitis B core antibody, HBeAb = hepatitis B e antibody, HBeAg = hepatitis B e antigen, HBsAb = hepatitis B surface antibody, HBsAg = hepatitis B surface antigen, HBV DNA = hepatitis B virus deoxyribonucleic acid, HDL = high-density lipoprotein, HVPG = hepatic venous pressure gradient, LDL = low-density lipoprotein, N = neutrophils, PLT = platelet count, RBC = red blood cell count, TBIL = total bilirubin, TG = triglycerides, WBC = white blood cell count.

### 2.2. Case 2

A 62-year-old male with a 5-year history of hepatitis B cirrhosis had been receiving long-term antiviral therapy with entecavir dispersible tablets. On admission, vital signs were: temperature 36.2°C, pulse 49 beats per minute, blood pressure 115/82 mm Hg. He was alert, with a chronic liver disease facies and liver palms, no spider angiomas, no jaundice in the skin or sclera, normal cardiopulmonary auscultation, soft abdomen without tenderness or rebound pain, positive hepatic percussion tenderness, no hepatosplenomegaly on palpation, and negative shifting dullness.

#### 2.2.1. Laboratory and imaging findings

HBV DNA: <5.00 × 10^2^ IU/mL; HBsAg: 1272.76 IU/mL; anti-HBs: 0 mIU/mL; HBeAg: 0.37 s/co; anti-HBe: 0.18 s/co; anti-HBc: 6.05 s/co.

Liver function: total bilirubin 21.7 μmol/L, direct bilirubin 7.6 μmol/L, indirect bilirubin 14.1 μmol/L, ALT 12 U/L, AST 15 U/L, GGT 20 U/L, albumin 45 g/L.

Renal function: UREA 5.4 mmol/L, CREA 77 µmol/L.

Fasting blood glucose: 5.2 mmol/L.

Myocardial enzyme profile: LDH 136 U/L, CK 49 U/L, CKMB 6 U/L, HBDB 117 U/L.

Abdominal ultrasound: cirrhosis, splenomegaly; transient elastography: CAP 217 dB/m, liver stiffness measurement (LSM) 12.3 kPa.

#### 2.2.2. Treatment course and follow-up

Entecavir therapy was continued, and HVPG measurement via antecubital vein catheterization revealed HVPG = 9.4 mm Hg. Due to sinus bradycardia (contraindication for carvedilol), simvastatin (10 mg orally once daily) was initiated. However, the patient self-discontinued simvastatin one week after discharge.

One year later, the patient presented with fatigue and abdominal distension. Laboratory and imaging results showed:

Virological and serological parameters: HBV DNA: <5.00 × 10^2^ IU/mL; HBsAg: 854.42 IU/mL; anti-HBs: 0.12 mIU/mL; HBeAg: 0.44 s/co; anti-HBe: 0.24 s/co; anti-HBc: 6.91 s/co.

Liver function: total bilirubin 23.2 μmol/L, direct bilirubin 10.2 μmol/L, indirect bilirubin 13.0 μmol/L, ALT 17 U/L, AST 17 U/L, GGT 25 U/L, albumin 44.1 g/L.

Renal function: UREA 4.86 mmol/L, CREA 77.2 µmol/L.

Fasting blood glucose: 4.77 mmol/L.

Myocardial enzyme profile: LDH 161 U/L, CK 87 U/L, CKMB 11.4 U/L, HBDB 112 U/L.

Abdominal ultrasound: unchanged cirrhosis and splenomegaly; transient elastography: CAP 208 dB/m, LSM 13.5 kPa; HVPG repeat measurement: 10.0 mm Hg.

Simvastatin (10 mg orally once daily) was restarted. After 3 months of treatment, follow-up showed:

Virological and serological parameters: HBV DNA: <5.00 × 10^2^ IU/mL; HBsAg: 690.39 IU/mL; anti-HBs: 0 mIU/mL; HBeAg: 0.41 s/co; anti-HBe: 0.25 s/co; anti-HBc: 6.42 s/co.

Liver function: total bilirubin 26.7 μmol/L, direct bilirubin 11.8 μmol/L, indirect bilirubin 14.9 μmol/L, ALT 21 U/L, AST 20 U/L, GGT 26 U/L, albumin 45.5 g/L.

Renal function: UREA 5.3 mmol/L, CREA 74 µmol/L.

Fasting blood glucose:5.1 mmol/L.

Myocardial enzyme profile: LDH 153 U/L, CK 78 U/L, CKMB 8 U/L, HBDB 113 U/L.

**Figure 2.**
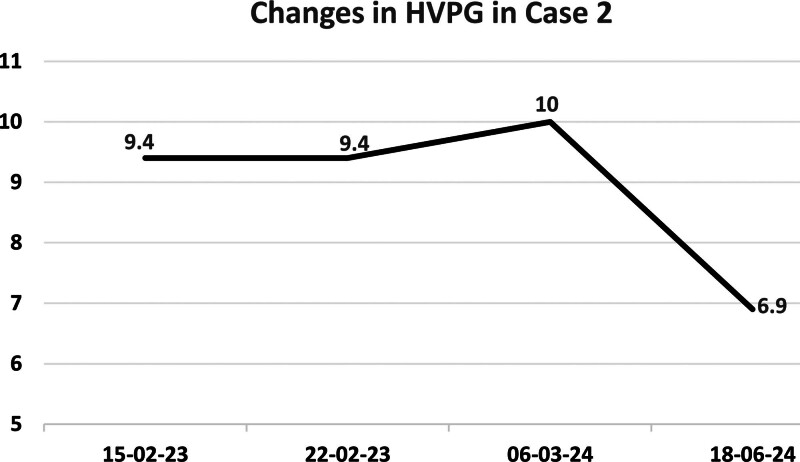
Changes in HVPG in case 2. HVPG = hepatic venous pressure gradient.

Abdominal ultrasound: stable cirrhosis and splenomegaly; transient elastography: CAP 179 dB/m, LSM 12.9 kPa; HVPG: 6.9 mm Hg, representing a 31.0% reduction from the second measurement (dynamic changes in HVPG shown in Fig. 2).

We continued to follow up with the patients after the third HVPG measurement, and the patients underwent reexamination of relevant indicators five months later.

Virological and serological parameters: HBV DNA < 5.00 × 10^2^ IU/mL; HBsAg 768.42 IU/mL; anti-HBs 0.96 mIU/mL; HBeAg 0.41 s/co; anti-HBe 0.30 s/co; anti-HBc 6.21 s/co.

Liver function: total bilirubin 18.1 μmol/L, direct bilirubin 8.5 μmol/L, indirect bilirubin 9.6 μmol/L, ALT 17 U/L, AST 18 U/L, GGT 21 U/L, albumin 40.7 g/L.

Renal function: UREA 6.59 mmol/L, CREA 73.1 µmol/L.

Fasting blood glucose: 4.81 mmol/L.

Myocardial enzyme profile: LDH 145 U/L, CK 80 U/L, CKMB 7.3 U/L, HBDB 114 U/L.

Imaging: abdominal ultrasound unchanged (cirrhosis); transient elastography: CAP 176 dB/m, LSM 12.5 kPa (laboratory changes listed in Table 2).

**Table 2:** Changes in laboratory findings of case 2.

	Laboratory tests before the first HVPG measurement	Laboratory tests before the second HVPG measurement	Laboratory tests before the third HVPG measurement	5 mo after the end of follow-up
HBsAg (IU/mL)	1272.76	854.42	690.39	768.42
HBsAb (IU/mL)	0	0.12	0	0.96
HBcAb (s/co)	6.05	6.91	6.42	6.21
HBeAg (s/co)	0.37	0.44	0.41	0.41
HBeAb (s/co)	0.18	0.24	0.25	0.30
HBV-DNA (IU/mL)	<5 × 102	<5 × 102	<5 × 102	<5 × 102
TBIL (µmol/L)	21.7	23.2	26.7	18.1
DBIL (µmol/L)	7.6	10.2	11.8	8.5
ALT (U/L)	12	17	21	17
AST (U/L)	15	17	20	18
ALP (U/L)	76	85	79	85
GGT (U/L)	20	25	26	21
ALB (g/L)	45	44.1	45.5	40.7
UREA (mmol/L)	5.4	4.86	5.3	6.59
CREA (µmol/L)	77	77.2	74	73.1
GLU (mmol/L)	5.2	4.77	5.1	4.81
LDH (U/L)	136	161	153	145
CK (U/L)	49	87	78	80
CKMB (U/L)	6	11.4	8	7.3
HBDB (U/L)	117	112	113	114
CHOL (mmol/L)	3.91	3.72	2.71	2.84
HDL (mmol/L)	1.09	1.17	1.15	1.08
LDL (mmol/L)	2.40	2.07	1.33	1.57
TG (mmol/L)	0.53	0.65	0.54	0.50
WBC (109/L)	2.64	3.51	3.51	2.79
N (109/L)	1.36	1.57	1.68	1.30
RBC (1012/L)	4.68	4.48	5.3	4.34
PLT (109/L)	48	51	45	54
CAP (dB/m)	217	208	179	176
E (kPa)	12.3	13.5	12.9	12.5

ALB = albumin, ALP = alkaline phosphatase, ALT = alanine aminotransferase, AST = aspartate aminotransferase, CAP = controlled attenuation parameter, CHOL = total cholesterol, DBIL = direct bilirubin, E = liver stiffness measurement, GGT = gamma-glutamyl transferase, HBcAb = hepatitis B core antibody, HBeAb = hepatitis B e antibody, HBeAg = hepatitis B e antigen, HBsAb = hepatitis B surface antibody, HBsAg = hepatitis B surface antigen, HBV DNA = hepatitis B virus deoxyribonucleic acid, HDL = high-density lipoprotein, HVPG = hepatic venous pressure gradient, LDL = low-density lipoprotein, N = neutrophils, PLT = platelet count, RBC = red blood cell count, TBIL = total bilirubin, TG = triglycerides, WBC = white blood cell count.

## 3. Discussion

Portal hypertension in cirrhosis refers to a clinical syndrome caused by increased pressure in the portal vein and its tributaries due to impaired portal blood flow or increased blood volume, occurring on the basis of cirrhosis.^[7]^ The etiologies of cirrhosis are complex and diverse, with chronic hepatitis B virus infection being the primary cause in China.^[8]^

Antiviral therapy is the cornerstone of treatment for hepatitis B-related cirrhosis.^[9]^ However, even with effective antiviral therapy, a significant number of cirrhotic patients progress from the compensated to decompensated stage,^[10]^ with portal hypertension serving as a critical independent risk factor in this process.^[11,12]^

The effective management of portal hypertension remains a key challenge in cirrhosis treatment. Hepatic venous pressure gradient (HVPG) is the internationally recognized gold standard for assessing portal pressure.^[1,11]^ In the cases reported here, HVPG measurement was used to confirm the diagnosis of clinically significant portal hypertension, and post-treatment HVPG monitoring allowed direct and accurate evaluation of simvastatin’s therapeutic efficacy.

Carvedilol is a mainstay medication for portal hypertension,^[13]^ reducing HVPG through multiple mechanisms, preventing variceal bleeding, and improving patient outcomes. However, adverse effects such as bradycardia, hypotension, and acute kidney injury limit its use in some patients.^[2]^

Simvastatin, a natural hydroxymethylglutaryl-coenzyme A (HMG-CoA) reductase inhibitor, has been used as a first-line medication for dyslipidemia for decades. Recent studies have highlighted its potential in reducing portal hypertension. It lowers portal pressure in cirrhotic patients through mechanisms including inhibition of the p38 mitogen-activated protein kinase (p38 MAPK) signaling pathway, upregulation of endothelial nitric oxide synthase (eNOS) expression and activity in umbilical vein endothelial cells, increased Krüppel-like factor 2 (KLF2) expression, promotion of nitric oxide (NO) release, and reduction of intrahepatic vascular resistance to improve hepatic perfusion.^[5]^ Additionally, simvastatin exhibits anti-fibrotic effects.^[14–16]^ Clinical studies, such as those by Zhang et al,^[17]^ compared simvastatin with propranolol in cirrhotic patients and found both drugs reduced portal vein and splenic vein diameters, indicating efficacy in lowering portal pressure. Zheng et al^[18]^ demonstrated that simvastatin decreases portal inflow resistance by increasing NO levels and alleviates hepatic fibrosis by reducing hyaluronidase and serum type III procollagen levels. While these studies suggest a role for simvastatin in improving cirrhotic portal hypertension, they lacked precise efficacy evaluation using the gold-standard HVPG measurement.

Both cases reported here underwent HVPG measurement before and after treatment. In Case 1, carvedilol was initially combined with antiviral therapy but was discontinued after 1 week due to significant bradycardia, and simvastatin was initiated. Seven months later, HVPG decreased by 23.2% compared to baseline. In Case 2, simvastatin was prescribed alongside long-term antiviral therapy due to borderline HVPG and bradycardia (contraindication for carvedilol), but the patient self-discontinued it after 1 week. One year later, a repeat HVPG showed an increase, prompting reinitiation of simvastatin. After 3 months of treatment, HVPG decreased by 31% compared to the second measurement, further supporting the benefit of simvastatin in improving portal hypertension. Meanwhile, during treatment, the indicators including myocardial enzyme profile, renal function, and fasting blood glucose of the 2 patients were all within the normal range, and no drug-related adverse reactions occurred.

In conclusion, antiviral therapy combined with simvastatin shows promise as an effective treatment for portal hypertension in hepatitis B cirrhosis, potentially filling the therapeutic gap for patients intolerant to carvedilol. These cases highlight the value of HVPG monitoring in evaluating treatment efficacy and emphasize simvastatin’s role as an alternative option in clinical practice.

## Author contributions

**Conceptualization:** Xiaolong Qi.

**Investigation:** Yong Zhou, Xuefang Li, Hui Wang, Zhensheng Liu.

**Methodology:** Wei Gou.

**Writing – original draft:** Haodong Zhang.

**Writing** – **review & editing:** Jinjin Li.
